# The Electro-Cardiograph: Laboratory Findings and Clinical Applications

**Published:** 1915-03-06

**Authors:** 


### THE ELECTRO-CARDIOGRAPH.

### Laboratory Findings and Clinical Applications.

Few things have been more conducive to the development of scientific medicine than the increased facilities for the co-ordination of laboratory research with clinical practice that are such a conspicuous feature of recent hospital improvements. It is, after all, only during the last two decades that funds have been at all readily forthcoming for that side of hospital work which is connected with scientific investigation, and for many years it was difficult to persuade a generous public that all moneys given to the hospitals ought not to be devoted to the immediate needs of sick persons. An example of the opportunities for clinical research available for the present-day student of medicine is to be found in cardiology, practically a new science which, developed at the hands of James Mackenzie and his school, now enables the workings of the heart in health, and its abnormalities of rhythm and beat in disease, to be graphically demonstrated in a way that was impossible to those who had to rely entirely on time-honoured methods of inspection, palpation, percussion, and auscultation for demonstration purposes. Cardiology has, indeed, brought into use a new set of extremely delicate apparatus that presents to the student particularly interested in diseases of the circulation an additional sense organ, just as the *x-ray* apparatus has given a sixth sense of the utmost practical value to the surgeon. To-day our leading cardiologists are to be found working in specially equipped rooms connected with hospital wards, but it must not be forgotten that the early experiments on which much of their present work has been based were carried out in physiological laboratories far removed from clinical departments, and so under obvious disadvantages. Moreover, there has perhaps been some danger lest in the enjoyment of the fruits of victory the importance of these primitive experiments may be forgotten.

Consequently, special interest attached to the lecture given by Professor A. D. Waller, F.R.S., of London University, at a special meeting of the St. Mary's Hospital Medical Society last week (February 24), when the subject dealt with was "The Electrical Action of the Human Heart in 1887 and in 1915." Going back some thirty years, Professor Waller described his first observations of the important fact that, by connecting one arm and one leg of a normal man to a delicate galvanometer, it was possible to demonstrate the minute electrical effects produced by the heart's beat; emphasising his delight when, having demonstrated that a certain effect occurred in regard to a healthy individual, he proved his point by obtaining a reversed effect in the case of an individual whose heart happened to be transposed. This early experiment in the St. Mary's physiological department, carried out, as it was, with a simple type of capillary electrometer, presaged the modern electro-cardiograph, which, by revealing delicate electrical changes, tells the clinician much more nowadays. In the course of his remarks, Dr. Waller referred to several points affecting ward work, and drew attention to various conclusions which have an important bearing on hospital routine. Thus he pleaded for a simpler nomenclature in reference to results obtained by modern cardiological instruments, and deplored the tendency to multiply terms and devise an increasing number of polysyllabic words noticeable in some writings on this subject. Admittedly the inflation of technical terminology adds to the labours of the practical worker, and, consequently, by using the simplest possible nomenclature, a saving of time can be effected which is, of course, in the interest of institution, patients, and physicians. Certainly he himself had no difficulty in giving explanations of complex heart phenomena in very simple language, such as could be readily understood by anyone but slightly versed in the matters discussed. Then, again, the lecturer suggested that a great saving of time in reference to heart problems might be brought about in the clinical laboratory by a closer study of the electrical reaction obtained from normal hearts—that is to say, that the spending of a little more time in the physiological laboratory by those beginning the study of cardiology to-day would save them much unnecessary expenditure of time and energy when working in the wards later on. For it must be borne in mind that Professor Waller has demonstrated that, under certain circumstances, with the aid of the electro-cardiograph, in the case of healthy persons tracings have been obtained resembling those cardiograms secured on the clinical side with diseased hearts. This being so, it follows that, unless the importance of this physiological research is appreciated, mistakes in diagnosis are likely to be made by the cardiologist who has based his studies too largely on conditions found in the wards. It appears that the human heart in health tends to vary in its actual position within the chest in such a way that two distinct types can be recognised, and Dr. Waller showed radiograms illustrating this. To put the matter briefly, the heart may approximate either to the horizontal or to the vertical position, and its variation in this respect is accountable for differences in tracings obtained from different persons.

In short, after carrying out a series of researches of first-rate importance during the period 1887 to 1915, Professor Waller concludes that the electrical reactions of the human heart, as expressed graphically by the best apparatus that has been installed in the hospitals, do not reveal all that some workers have claimed for the method. On the other hand, he considers that in the detection of certain irregularities of rhythm, particularly of small extra beats—extra-systoles—the electro-cardiograph is of great value. Apropos of this he referred to the problem of heart failure in the early stages of chloroform anæsthesia, and suggested that it might be possible to obtain some warning of impending disaster by detecting such extra-systoles, for which purpose the patient would, of course, have to be connected up to the necessary instrument. Inasmuch as this connection can be effected by attaching a wire to one wrist and another wire to one ankle of a person about to be anæsthetised, and leading these wires off to an electro-cardiograph in another room, it is evident that such an observation could be made without any disturbance or discomfort to the patient. Herein is suggested an important and promising line of research in which the application of cardiological methods to anæsthetic problems may possibly free the anæsthetist from that bogey of sudden heart failure which is always present even with the most expert administrators when giving chloroform. It is noteworthy that Professor Waller has not only urged the younger men at St. Mary's Hospital to take up this work, but has offered to the anæsthetic department there the loan of an electro-cardiograph, and the support of his unrivalled experience in analysing the results obtained.

